# Rapid development of an evidence- and consensus-based guideline for controlling transmission of SARS-CoV-2 in schools during a public health emergency – A process evaluation

**DOI:** 10.3389/fpubh.2023.1075210

**Published:** 2023-03-30

**Authors:** Katharina Wabnitz, Mike Rueb, Lisa M. Pfadenhauer, Brigitte Strahwald, Eva A. Rehfuess

**Affiliations:** ^1^Institute for Medical Information Processing, Biometry and Epidemiology (IBE), Chair of Public Health and Health Services Research, Ludwig-Maximilians-Universität München, Munich, Germany; ^2^Pettenkofer School of Public Health, Munich, Germany

**Keywords:** public health guideline, rapid guideline, WHO-INTEGRATE, Evidence-to-Decision framework, evidence, expertise

## Abstract

**Introduction:**

Different measures to prevent and control the spread of SARS-CoV-2 have been implemented in German schools. Decisions regarding such measures should be informed by evidence regarding their effectiveness, and their unintended consequences for health and society. A multi-stakeholder panel was convened to develop an evidence- and consensus-based guideline for school measures, using the novel WHO-INTEGRATE framework. Developing a guideline to inform decision-making outside of the clinical realm during a public health emergency was unprecedented in Germany. This study aims to identify lessons learnt for similar endeavours by addressing the following research question: What were the strengths and weaknesses of the guideline development process as perceived by the different groups involved?

**Methods:**

Fifteen semi-structured interviews were conducted virtually. We recruited participants aiming to include the perspectives of all groups contributing to the guideline development, including both panel members (scientists, practitioners, school family and observers) and the guideline secretariat. For analysis, we carried out deductive-inductive thematic qualitative text analysis according to Kuckartz, structuring findings using a category system.

**Results:**

Due to time pressure, the guideline secretariat was heavily involved not only in synthesising the evidence but also in developing and drafting recommendations. Participants critically reflected on certain methods-related decisions, including the development of draft recommendations and application of the WHO-INTEGRATE framework by scientists only. The full potential of the framework might not have been harnessed. Participants’ understanding of relevant and valid evidence varied, and the available evidence base was limited. Participants represented different types of expertise, notably expertise informed by scientific evidence and expertise grounded in lived experience, influencing their involvement in the guideline development process and discussions during meetings.

**Conclusion:**

Developing an evidence- and consensus-based public health guideline in only three months was challenging, notably because of the involvement of a broad range of stakeholders and the use of a novel Evidence-to-Decision framework, both unprecedented in Germany. Learning from this process with a view to “institutionalising” the development of public health guidelines and refining methods can contribute to more evidence-informed public health decision-making in Germany and beyond, in general and during a public health emergency.

## Introduction

During the COVID-19 pandemic, public health and social measures (PHSM), also referred to as non-pharmacological interventions, have been implemented around the world across a range of settings. Measures to prevent and control the spread of SARS-CoV-2 in schools (ff. school measures) include interventions to reduce the opportunity for contacts such as cohorting; measures to make contacts safer such as mask mandates and surveillance and response measures such as testing (1). The effects of these measures on SARS-CoV-2-related health outcomes are likely contingent on levels of community transmission and on which other PHSM are implemented in the community (2). Yet, school measures can also lead to a broad range of unintended and often negative consequences, including anxiety and decreased wellbeing, skin reactions to masks or disinfectants, impaired educational attainment (3) and reduced opportunities for income generation of households with students in quarantine (4).

To enable safe and continuous school operations during the COVID-19 pandemic and to provide decision-makers and politicians with a scientific basis for their decisions, delegates from across a range of scientific societies, and from organisations representing students, teachers and parents (ff. school family) as well as public health practitioners, set out to develop an evidence- and consensus-based guideline for school measures (5). The guideline panel thus included a broad range of perspectives, notably of those assessing the impacts of school measures through multiple disciplinary lenses, of those implementing these measures and of those directly or indirectly affected by them. The aim was to render this a “living guideline” which would be regularly updated for the recommendations to remain valid in light of the constantly evolving pandemic context and the rapidly changing evidence base. The inclusion of all stakeholders targeted or affected by a guideline’s recommendations is an integral part of established quality criteria such as *The Appraisal of Guidelines for Research & Evaluation Instrument (AGREE II)* (6).

In Germany, the Association of the Scientific Medical Societies (Arbeitsgemeinschaft für Medizinisch-Wissenschaftliche Fachgesellschaften, AWMF) coordinates the development of clinical guidelines in adherence with the AGREE II quality criteria. These are classified according to the extent to which “elements of systematic development” (7) are applied, with the label “S3” referring to the highest level in the AWMF classification of guidelines. This requires systematic searches for evidence and an assessment of the quality of this evidence, as well as structured consensus-building and the representation of affected groups on the panel. The S3-guideline for the prevention and control of SARS-CoV-2 transmission in schools (ff. S3-guideline) was initiated in November 2021 in the context of the COVID-19 Evidence Ecosystem (CEOsys) project (8) and scientifically led and coordinated by the Chair of Public Health and Health Services Research at LMU and supported by the AWMF (ff. guideline secretariat). A first short version of the guideline was published in February 2021; this was subsequently refined and published as a long version in November 2021. The actual development of recommendations by dedicated working groups and voting on these by the guideline panel took place prior to February 2021. Table 1 presents an overview of key methodological steps of the guideline development process, adapted from AWMF procedures. Topic areas for which recommendations were to be developed were prioritised through consensus voting (see Table 2).

**Table 1:** Key methods-related steps of developing the first short and long versions of the S3 guideline in 2020 and 2021.

Timeline	Key methods-related steps	Actors involved
26th November 2020	Formal registration of the guideline with the AWMF by three scientific member societies and the German Scientific Society for Public Health	German Society of Pediatrics and Adolescent Medicine
		German Society of Epidemiology
		German Society for Pediatric Infectious Diseases
		German Scientific Society for Public Health
From 26th November 2020	Stakeholder mapping of potential panel member institutions with a view to achieving diversity in federal states (North, South, East and West) and municipalities (urban, rural). In selecting panel member institutions, the guideline secretariat aimed for balance between actors from the education and health sectors as well as affected groups and actors tasked with the practical implementation of measures (e.g. local health authorities)	Guideline secretariat
From 30th November 2020	Formal invitation of further scientific societies and organisations according to the results of the stakeholder mapping	Guideline secretariat
9th–16th December 2020	Literature search for sources of direct^1^ evidence as part of the Cochrane Rapid Review on the effectiveness of non-pharmacological measures to prevent and control SARS-CoV-2 transmission in schools [submission of protocol 2nd December 2020 which was informed by a scoping review (searches conducted 10th October 2020)]	Guideline secretariat
1st–13th December 2020	Online survey to prioritise relevant research questions for the school guideline amongst all members of the guideline panel	Guideline secretariat
16th December 2020	Constituting meeting of the guideline panel and secretariat Introduction of panel members Presentation of methodological steps of guideline development Presentation of WHO-INTEGRATE framework Consensus on the research questions	Guideline panel and secretariat
5th January 2021	Literature searches in six databases for sources of indirect^2^ evidence	Guideline secretariat
5th January 2021	Meeting of the secretariat with registering scientific societies Presentation of research questions according to the PICO (Population, Intervention, Control, Outcome) framework based on topics that were formally included in the guideline Formation of working groups, consisting of members of scientific associations to develop recommendations for each research question based on the PICO questions and results from direct and indirect evidence searches	Guideline secretariat
		German Society of Pediatrics and Adolescent
		Medicine
		German Society of Epidemiology
		German Society for Pediatric Infectious Diseases
		German Scientific Society for Public Health
11th January 2021	Meeting of the secretariat with working groups Provision of direct and indirect evidence regarding each PICO question Provision of template to apply WHO-INTEGRATE framework	Guideline secretariat
		German Society of Pediatrics and Adolescent
		Medicine
		German Society of Epidemiology
		German Society for Pediatric Infectious Diseases
		German Scientific Society for Public Health
		German Society for Hygiene, Environmental and Preventive Medicine (*own translation*)
11th – 17th January 2021	Development of draft recommendations by working groups, including application of WHO-INTEGRATE framework	Guideline secretariat
		German Society of Pediatrics and Adolescent
		Medicine
		German Society of Epidemiology		
		German Society for Pediatric Infectious Diseases
		German Scientific Society for Public Health
		German Society for Hygiene, Environmental and Preventive Medicine (*own translation*)
14th January 2021	Meeting of scientific secretariat with working groups Discussion and consensus regarding classifications of age groups and rates of infection Interim reporting of small working groups on progress in recommendation development Planning of next full guideline group meeting	Guideline secretariat
		German Society of Pediatrics and Adolescent Medicine
		German Society of Epidemiology
		German Society for Pediatric Infectious Diseases
		German Scientific Society for Public Health
		German Society for Hygiene, Environmental and Preventive Medicine (*own translation*)
17th–19th January 2021	Online commenting and feedback on the draft recommendations by the guideline panel	Guideline panel and secretariat
19th of January 2021	Second meeting of the guideline panel and secretariat Consensus-building regarding draft recommendations was postponed following requests from the guideline panel for more time for review of draft recommendations and comment	Guideline panel and secretariat
28th and 29th of January 2021	Third and fourth meeting of guideline group Consensus-building regarding draft recommendations	Guideline panel and secretariat
8th February 2021	Publication of short version 1.0 of the guideline	AWMF
16th February 2021 and 3rd March 2021	Meetings of scientific secretariat with working groups Discussion of reception of guideline by decision-makers and uptake in the media Discussion of next steps regarding the long version of the guideline	Guideline secretariat
		German Society of Pediatrics and Adolescent
		Medicine
		German Society of Epidemiology
		German Society for Pediatric Infectious Diseases
		German Scientific Society for Public Health
		German Society for Hygiene, Environmental and Preventive Medicine (*own translation*)
23rd June 2021	Meeting of scientific secretariat with working groups Agreement to publish a press statement on remaining validity of the short version 1.0 Agreement on timeline for consensus-voting on changes to recommendations with the full group and publication of long version Discussion of procedure for updating the short version	Guideline secretariat
		German Society of Pediatrics and Adolescent
		Medicine
		German Society of Epidemiology
		German Society for Pediatric Infectious Diseases
		German Scientific Society for Public Health
		German Society for Hygiene, Environmental and Preventive Medicine (*own translation*)
2nd July 2021	Press release to confirm ongoing validity of the guideline version 1.0	Guideline secretariat
16th – 26th July 2021	Update of guideline to short version 1.1 *via* online consensus-voting by panel members	Guideline panel and secretariat
28th July 2021	Fifth meeting of guideline panel and secretariat Voting on suggested changes to two recommendations. Due to disagreements within the panel regarding the certainty of the available evidence, voting on these recommendations was postponed	Guideline panel and secretariat
11th – 22nd September 2021	Online consensus-voting on recommendation 7.2 and 9 by panel members	Guideline panel and secretariat
3rd – 17th November 2021	Adoption of the guideline by all panel members	Guideline panel and secretariat
26th November 2021	Publication of long version of the guideline	AWMF

Direct evidence: evidence on effectiveness of PHSM in the school setting, retrieved through: the Cochrane Rapid Review on the effectiveness of non-pharmacological measures to prevent and control SARS-CoV-2 transmission in schools (1) and further relevant systematic reviews identified through background searches during protocol development, searches for reviews and evidence syntheses in the WHO COVID-19 database, forward and backward citation screening through google scholar. Indirect evidence: evidence on effectiveness of PHSM in other settings than schools or with different population groups, retrieved through: screening of PubMed, Cochrane Special Collection COVID-19 and WHO COVID-19 database for relevant systematic reviews, reviews, evidence syntheses, forward citation searching through google scholar.

**Table 2:** Prioritised topic areas for the development of recommendations.

Cohorting of students and teachers, reducing the number of students at school
Wearing of face coverings by students and teachers
Measures for infection prevention on the way to school
Measures for infection prevention during physical education and music lessons
Attending school when symptoms of common cold are present in students or teachers
Quarantine rules for students and teachers
Ventilation, air purification and reduction of aerosol concentrations

**Figure 1:**
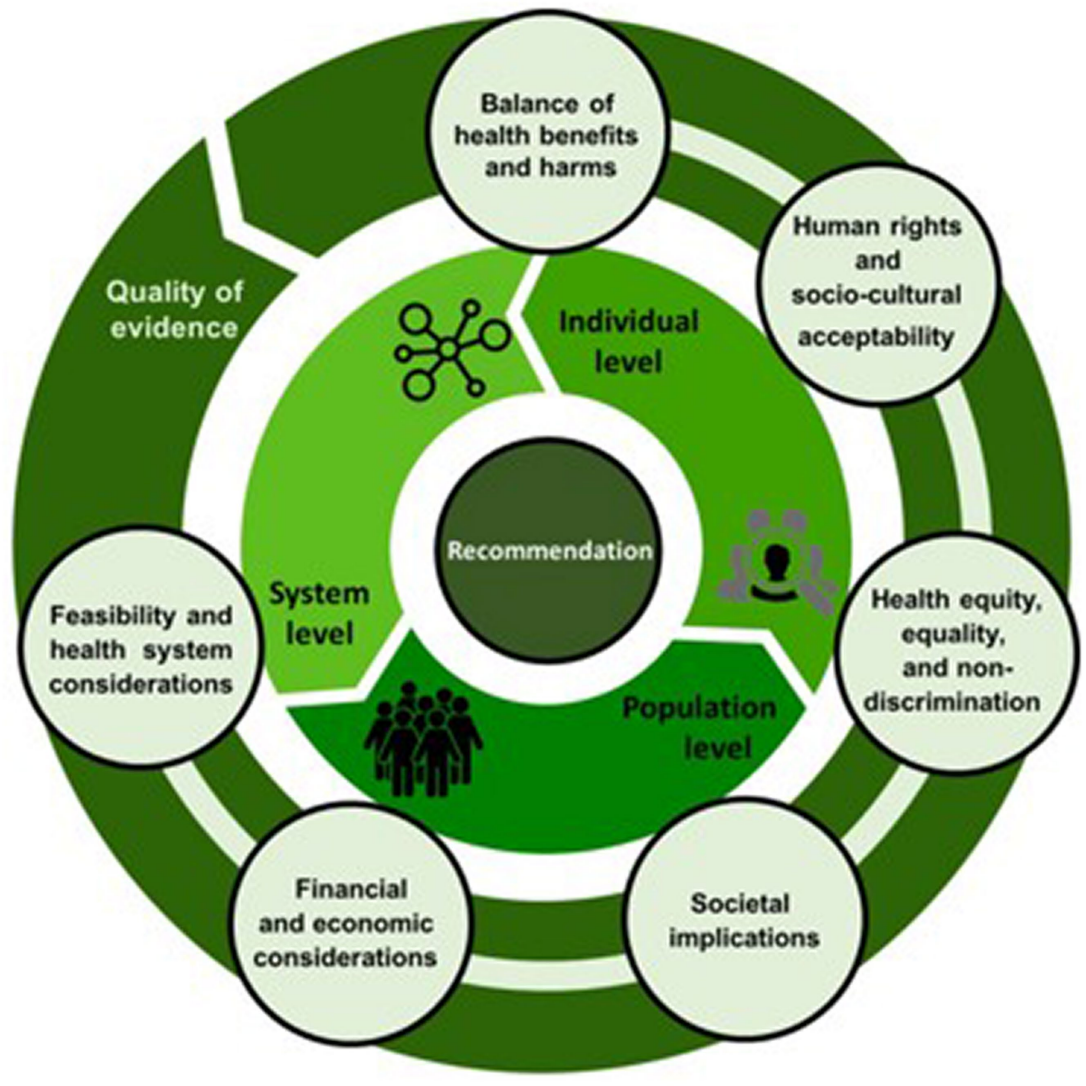
The WHO-INTEGRATE Evidence-to-Decision framework version 1.0 (9).

Developing an S3-guideline to inform decision-making outside of the clinical realm during a public health emergency was unprecedented in Germany. Based on an understanding of schools as complex systems and of any intervention to prevent or control SARS-CoV-2 infections taking place within such systems, a consideration of the potential effects of these interventions beyond health outcomes was emphasised during guideline development. Consequently, the WHO-INTEGRATE Evidence-to-Decision framework (EtD), developed with a view to aid decision-making “about complex interventions implemented in complex systems” (9), was applied during guideline development and—to our knowledge—for the first time in Germany. It proposes that six criteria plus quality of evidence be considered when formulating recommendations. These criteria are: balance of health benefits and harms, human rights and sociocultural acceptability, health equity, equality and non-discrimination, societal implications, financial and economic considerations and feasibility and health system considerations (Figure 1). A balanced consideration of these criteria from across multiple perspectives was sought through the inclusion of a broad stakeholder group, including members of the school family, in the guideline panel, as described above.

It is important to have a thorough understanding of the usefulness and impact of different processes and instruments seeking to aid evidence-based decision-making, especially in the context of the COVID-19 pandemic, which has required timely and potentially incisive measures. The S3-guideline represents such an instrument and evaluating its impact as well as the strengths and weaknesses of its development process can contribute novel insights to the research fields of evidence-based policymaking, as well as help identify lessons learnt and develop best practices for future health emergencies.

### Aim

This study represents one sub-study of a multi-pronged approach to evaluating the S3-guideline as an instrument for providing evidence-based support for decision-making. The focus of the present sub-study is on the guideline development process from the first constitutional meeting on 16th December 2020 until publication of the first long guideline version on 26th November 2021, notably it addresses the strengths and weaknesses of the guideline development process as perceived by the different groups involved. The impact of this guideline on political and practical decision-making as well as experiences with implementing measures in schools according to this guideline are not the subject of the present paper but the subject of further studies.

## Methods

### Design

We chose a qualitative research approach as we were interested in the subjective experiences and perspectives of those who participated in the guideline development process (10). We had to rely on a retrospective design given the time constraints during the guideline development process, which did not allow for planning and carrying out concomitant evaluative research. In our reporting, we follow the consolidated criteria for reporting qualitative research (COREQ) checklist (Supplementary Annex 3) (11).

### Sampling and recruitment

To ensure representation of all relevant perspectives, we categorised members of the guideline panel in four groups plus the guideline secretariat. We sent invitations to all members (*n* = 61) of the guideline panel and secretariat and informed them about the purpose of the study. Willingness to participate was expressed by the following number of participants within each of the five groups: (i) 9 scientists (of 23 invited; 1 declined, 13 did not respond), (ii) 4 public health practitioners (of 9 invited; 1 declined, 4 did not respond), (iii) 4 members of the guideline secretariat (of 8 invited; 0 declined, 4 did not respond, 1 participated in a pilot interview), (iv) 6 members of the school family (of 14 invited; 2 declined, 6 did not respond) and (v) 1 observer (of 6 invited; 0 declined, 5 did not respond). The only inclusion criterion was having served as a member of the guideline panel or guideline secretariat during the period indicated above. To choose amongst those who expressed willingness to participate in each group, we used an online random generator. In each group, we aimed to interview at least two individuals and included further participants according to the randomly generated order, with particular attention paid to achieving saturation regarding the perspectives of teachers, parents and students from within the school family group. The only exceptions to this approach concerned the representative of the AWMF as well as the pilot interview participant who we explicitly invited to participate and categorised as guideline secretariat.

### Data collection

All prospective participants were provided with study information sheets and consent forms. Two researchers (KW, MR) conducted semi-structured interviews in German from 12th November to 22nd December 2021, using an interview guide which contained open-ended questions in relation to the study aim (see Supplementary Annex 1). This was tested in a pilot interview with one member of the guideline secretariat and refined accordingly. Interviews were conducted *via* a web-based tele-conferencing tool. Questions concerned the following topic areas: (a) participants’ perception of the process, (b) their understanding of evidence and of the role of evidence in the process, (c) their understanding of expertise and of the role of expertise in the process and (d) views on the consideration of societal implications and unintended consequences. We also investigated participants’ views on the impact of the guideline on political decision-making, on lessons learnt for decision-making in public health (emergencies) as well as roles and communication during the guideline development process, which are not reported in this article. The interviews were recorded using a linear PCM recorder (OLYMPUS LS-P1). KW and MR independently wrote memos following each interview. All data were securely stored on an encrypted device and anonymised, with KW and MR having exclusive access to the primary, non-anonymised data to maintain confidentiality given the other authors’ active involvement in the guideline development process.

### Data analysis

Transcription of all interviews was carried out by Audiotranskription (12), a German external transcription service complying with data protection requirements. All audio files were destroyed after transcription. Each interviewee was provided with their anonymised transcript for review (member check). Any requests for further anonymisation or removal of potentially compromising data by interviewees were resolved (KW, MR). All transcripts were analysed using the qualitative analysis software MAXQDA (Version 11.4.1) (13). An active process of reflecting about the interviewers’ positionality (KW, MR) accompanied data collection, analysis and write-up. Data analysis was undertaken using the deductive-inductive approach of thematic qualitative text analysis according to Kuckartz (14). This method was chosen for its highly systematic approach and explicit use of deductive and inductive elements which allows for the evaluation of specific aspects of interest whilst leaving room for additional topics to be elicited. Using this approach, the following steps were undertaken: (1) familiarisation with the data (KW, MR); (2) deductive development of main thematic categories based on the topic guide (KW, MR); (3) independent coding of all transcripts using the main categories with subsequent comparison of results and resolution of conflicts through discussion and refinement of the main coding system, where needed (KW, MR); (4) inductive development of sub-categories within each main category (KW, with intra-coder reliability established through various rounds of applying the sub-categories to the data as well as discussion with MR and refinement where necessary); (5) analysis of the structured content within and across main categories (KW with input from all authors). The final category system (see Supplementary Annex 2) as well as the quotes presented below were translated into English by KW and checked by the other authors.

### Ethical approval

Ethical approval for this study was obtained prior to recruitment of participants from the Ethics Committee of the Medical Faculty, Ludwig-Maximilians-Universität München (No. 21-0944).

## Results

In total, 15 members of the guideline panel and secretariat were interviewed. Table 3 provides an overview of all interview participants according to their role in the guideline development process. Findings are structured according to the four main categories of the interview guide, including the inductively developed sub-categories within each of these main categories.

**Table 3:** Overview of study participants* and interview duration.

ID	Group	Duration of interview in minutes
B1	Guideline secretariat	40
B2	Guideline secretariat	43
B3	Guideline secretariat	41
B4	Scientist	32
B5	Scientist	41
B6	Scientist	26
B7	Guideline secretariat	50
B8	Scientist	54
B9	Public health practitioner	56
B10	Public health practitioner	40
B11	School family	29
B12	School family	46
B13	School family	34
B14	School family	27
B15	Observer	33
Total: 15		Total: 592

*No further characteristics provided to maintain confidentiality.

### Perception of the guideline development process

#### Methods and implementation of the guideline development process

The secretariat was described as “the structural guards of the guideline” (B5, scientist). Members of the secretariat were heavily involved in the guideline development process, not just in a coordinating and methods-related supporting role but also in helping with drafting and revising recommendations. Whilst one participant pointed out that “actually I understand that a secretariat always assists in guideline [development] […]. And that decisions are then made separately” (B8, scientist), it was also noted “that a lot was then left up to the staff [of the secretariat]” (B3, guideline secretariat). Even more explicitly, this participant said: “And later I also directly worked on the recommendations and supported the [working] groups. But partly I also wrote a lot myself” (B1, guideline secretariat).

Participants emphasised that they appreciated the transparent, democratic and anonymous nature of the consensus-building procedures. They also perceived it as useful and time-efficient that the scientific literature was being systematically identified and appraised for them:

“I found the scientific preparation VERY good. [..] For us as participants in the guideline panel, everything was prepared and presented very well and in a comprehensible manner” (B9, public health practitioner).

The iterative process of working in smaller groups and consensus voting with the full panel was generally described as efficient and goal-oriented. One member of the school family pointed out that they perceived the whole panel to be committed to the shared goal of keeping schools open and maintaining the best possible education even under pandemic circumstances. However, the process also involved some polarisation within the panel:

“[There were] players [..] who [..] suddenly put in a veto and said ‘We're definitely not going to go along with this and we have to start everything from scratch again’” (B6, scientist).

Certain methods-related decisions taken during the guideline development process were critically reflected upon by participants. One participant reported that, after voting on a few options for recommendations or wordings, the option with the highest agreement was immediately chosen instead of voting again on that option; this participant pointed out that the recorded votes may “not depict what a final voting might have resulted in” (B9, public health practitioner). Furthermore, not having formally prioritised endpoints for outcomes of interest to consider during the process was pointed out as a challenge by several participants, for example:

“We did not undertake a formal prioritisation of the endpoint[s]. [..] So we kind of presented and discussed them in the kick-off meeting […]. But we did not formally prioritise them” (B3, guideline secretariat).

One participant (B7, guideline secretariat) was particularly vocal about methods-related decisions and their implementation. For this analysis, this perspective ought to be seen as unique due to a particular interest and expertise in processes of evidence-based decision-making. One aspect criticised was the very high number of topic areas for recommendations, which in this person’s view deprived the panel of time and resources to discuss fewer recommendations in a more comprehensive manner. Similarly, the same participant noted that allocating more time to a thorough and comprehensive process of prioritising and then adapting the generic criteria of the WHO-INTEGRATE framework at the beginning of the process might have been beneficial, as using the generic criteria “later led to the fact that we simply needed a lot of coordination again, the teams needed a lot more support because they could not do anything with a lot of the stuff […] if we had simply stopped [earlier] at: Which endpoints are important to us? What should we look at through INTEGRATE sub-criteria? […] We would have saved a lot more time in the course [of the guideline development process]” (B7, guideline secretariat). This was partially echoed by another participant who said that they would have wanted to “actually go through these criteria again in detail, see if we have evaluated them correctly? Are there any problems in the area of implementation, for example, which should also inform the formulation of the guideline? And THAT could not take place, due to the time pressure” (B2, guideline secretariat). This participant even suggested that the assessment of criteria was based on a few individuals’ opinions:

“de facto, we did not make enough room for this process [consideration of societal implications], or perhaps we did not have the tools to do it particularly well. So we actually WANTED to attach so much importance to these criteria, but DIDN’T. Because two or three people somehow simply came up with what they thought about these criteria” (B1, guideline secretariat).

Similarly, it was mentioned that “the point about conformity with fundamental and human rights was like a box that had to be ticked once, […] yes or no. Yes, that wasn‘t a question for discussion at all” (B8, scientist), pointing to further aspects which influenced the application of the WHO-INTEGRATE framework as discussed in more detail under Consideration of societal implications and unintended consequences.

Furthermore, some participants perceived the composition of the guideline panel as imbalanced. In relation to this, the following comment regarding the extent to which legitimate representation was achieved is interesting:

“For example, it was not clear to me what criteria were used to include people or institutions from the education sector in the panel. I found that rather random. […] For example, the Federal Parents' Council is not strictly a participatory, democratically elected body […]” (B15, observer).

Methods-related decisions for the systematic review, which represented the main—although not the only—source of peer-reviewed evidence to inform the guideline development process, were also critically discussed. This concerned, for example, the decision to include modelling studies of varying degrees of quality alongside intervention studies based on empirical data, but to exclude basic research studies (e.g. lab-based studies). Consequently, a certain body of evidence was available and considered by the panel and this “did also frame the process, when saying ‘Here we have some evidence. Here we have no evidence’” (B7, guideline secretariat).

During the interviews, it emerged hat “The group was, so to speak, […] divided into those who had already thought things through, pre-formulated things in the specific working groups. And then, so to speak, as a reflecting team, then us as school experts” (B12, school family). This points to a procedural decision regarding participants’ distinct roles in the process, evoking diverging opinions. One participant’s comment shows that some may have perceived this approach as derogatory:

“So [the parents] were basically included […] in the formulation, but not in the development [of recommendations]. There were only a few institutions. For instance, the parents are not considered […] as experts” (B11, school family).

It was also questioned whether certain issues (e.g. feasibility or acceptability) might have been addressed differently, if members of the school family had been involved in the development of recommendations from the very beginning. Contrastingly, another participant questioned the value of having debates on an eye-level between all guideline panel members:

“But I dare to doubt whether all people then have to vote on everything on equal terms. So to put it very concretely, it has sometimes been difficult for me to discuss with people what the scientific evidence in favour or against a recommendation is. And conversely, as a scientist, I don't want to make presumptions about the implementation in practice. […] involving them [those affected by or tasked with implementation of measures] right at the beginning […] I found difficult and tedious” (B8, scientist).

Lastly, it was pointed out that the guideline fell short of considering how the recommendations would interact with each other and whether following through on some of them might render others redundant, e.g. whether a mask mandate would still be warranted if distancing and ventilation measures were fully implemented.

#### Guideline development under time pressure

Operating under time pressure was identified as a big challenge by participants. As drafts for the recommendations were developed by small working groups only, these might have been perceived as a “done deal” (B2, guideline secretariat) because: “It was certainly the case, and we also regretted that […] there wasn’t enough time to explain everything in detail to the others” (B6, scientist). This suggests that there was not enough time to fully address all concerns within the panel and to create a shared understanding of the rationale for recommendations before they were voted upon. This participant pointed out that “in my view, a more intensive debate was missing in the aftermath or in the whole implementation [of the process] […] And that takes time and therefore, given the dynamics of COVID, we simply do not have that time, so for me it is questionable to what extent such a guideline is purposeful” (B11, school family).

Sharing a lot of material for information and comment shortly before meetings with the panel “also caused discontent” (B1, guideline secretariat), as it constituted the only option for those who were not part of the working groups to familiarise themselves with and comment on the draft recommendations before voting on them. It was suspected that “given that this was roundabout a 100-page document, only very few people did that [reviewed the material]. Simply for capacity reasons” (B1, scientific secretariat). Related to this, participants stated that they felt overwhelmed with having too little time to prepare for the panel meetings. Slightly contrary to the general notion of time pressure, one participant noted that they had enough time to make up their mind about their voting decision: “These coordination processes were often such that one had time […] to think it through afterwards and to deliberate at home on the computer how I should decide now. […] So, in this respect, no one can really complain that they were somehow forced to make decisions too spontaneously, too suddenly” (B13, school family).

### Evidence and its role in the process

#### Understanding of scientific evidence

There were discrepancies in how scientific evidence was interpreted and perceived by different groups represented in the guideline panel, as well as the secretariat. Those with a background in epidemiology and public health emphasised a need to focus on population-level outcomes when developing recommendations for this guideline. They also mentioned other defining aspects for scientific evidence, notably the systematic consideration of all available evidence and the assessment of its quality, as well as a notion of usefulness and reliability of certain types of evidence compared to others. For members of the school family, the notion of scientific evidence as well as scientific methods to be applied to assess certain phenomena was probably inherently different from those with a background in science:

“I would say that perhaps scientific disciplines like medicine […] have an easier time. Because you can measure a lot of things quite clearly and then it's clear that I'll do so and so many studies with certain parameters and determine the evidence from them. This is much more difficult in the pedagogical field because, for example, you cannot work so much with control groups or with placebos […] So I think that the question of ‚based on evidence ‘[…] is perhaps not so common among people who work in the pedagogical field” (B13, school family).

It was also mentioned that certain types of evidence should have received more weight during the process, for example basic research studies:

“There were [..] different interpretations of what evidence really is, and of what direct and indirect evidence is. So, the majority […] they are epidemiologists somehow and they have very clear standards of what evidence means […] for me, evidence from basic research counted just as much […] and I would give that a little more weight […] than it actually happened in the group, because the very clear, hard epidemiological criteria were applied there” (B6, scientist).

Thus, the understanding of evidence and the extent to which certain types of evidence could or should be used to base conclusions on, appears to have varied between the different interview participant groups. Not only were there differences amongst those with a background in science but also between this group and members of the school family. For example, an understanding of the importance of standard scientific practice appears not to have been equally shared by all members of the guideline panel and secretariat:

“Especially among non-medical persons or [..] non-scientific persons who participated, […] they often simply sent us statements by some expert that somehow did not even list any references [..] Which for me would be a reason to simply exclude any studies from a guideline. And that was not accepted by others” (B8, scientist).

The following statement suggests that the separation of participants in those actively involved with drafting the recommendations and those commenting on the outputs could be linked to a different understanding of evidence and its role in the process:

“I suspect that the understanding varies greatly within the group. So those, especially the scientific societies, who developed the recommendations in close coordination with [the secretariat], I would assume they HAVE this understanding. I suspect that many of the others, who actually only came to the guideline meetings, do not have this distinction so clearly in their minds” (B2, guideline secretariat).

The following quote alludes to the importance of providing more opportunities to develop a shared understanding of evidence and its role:

“And there were some very strong misunderstandings at the beginning, which we, namely I, was able to resolve through conversation and also through an exchange about what evidence actually is. I found that very helpful. But these were background discussions, nothing that the whole group took part in” (B8, scientist).

#### Role of evidence in the process

In principle, participants agreed that evidence had to and did play a critical role in the process.

“a very big [role] and it also has to be that way at the AWMF […] That’s what the guidelines are based on” (B6, scientist).

Participants referred to the systematic review on the effectiveness of school measures (1) as the primary source of evidence for the guideline, which justified the classification of the guideline as an S3-guideline. Limitations to the role that evidence played in the guideline development process with supporting quotes are summarised in Table 4. Interestingly, one participant also questioned the usefulness of aspiring to have “evidence for everything”:

**Table 4:** Sub-category “Limitations to the role evidence could play in the process” with supporting quotes.

Limitations to the role evidence could play in the process	Supporting quotes
Lack of availability of directly relevant empirical studies	“One is that we […] do not actually have very well conducted empirical studies with good epidemiological studies, so to speak, in the pandemic. This means that we often fall back on modelling studies” (B2, guideline secretariat)
No consideration of qualitative evidence	“Qualitative evidence does not really come into play here, it has to be said. That’s just difficult in this process […] but you also have to see that this is also an important source of insight and information for public health” (B5, scientist)
Issues regarding the transferability of findings from specific populations/settings to the school setting	“While for mask-wearing, we had a systematic, yes a systematic review, which generally showed the usefulness of masks, there was a debate about whether this evidence was transferable to the context we were considering at all. Because at that time most of the studies were from hospitals” (B8, scientist)
Ethical and other feasibility issues regarding the generation of high-quality evidence during a pandemic	“So it’s ethically not feasible to say that we’ll somehow equip 50 per cent of the school classes here in the high-risk area with air purifiers and not the others, and then we’ll see what happens somehow. That could not have been done” (B6, scientist)
Differential insight into the evidence by individuals with different disciplinary backgrounds	“Especially with the people who wrote the recommendations. With them, I noticed very strongly that they always went back into the studies, looked again and then presented it in this way. I do not think that the other members re-read the studies themselves, but rather trusted that what is said by the people who make the recommendations is correct” (B8, scientist)
Decisions during the systematic review regarding inclusion and exclusion of studies	“And within the review, a series of decisions were made as to which evidence was taken into account and which wasn‘t. […] And that is this kind of methodological decision that led to the fact that for certain things there was evidence and for certain things there was no evidence. […] So S3 is simply based on this process: There was a review, but the evidence we have is just so bad that you can really ask yourself: Is this any different to an expert opinion?” (B7, scientific secretariat)
Most recent pandemic developments at the time of guideline development were not reflected in systematic review	“The second is that the available evidence is currently not up-to-date but actually we mainly still have studies until December 2020 at hand, so to speak, with all the problems that this causes: delta variant not represented, many studies not yet included or not yet systematically searched for and considered” (B2, guideline secretariat)
Lack of a shared understanding of the concept of relevant and valid evidence	“Based on that, this assessment of what is an evidence-based argument, or what is evidence, […] was an issue in the beginning to agree on what are hard facts for us. So, and that was not easy” (B12, school family)

“A disadvantage of the guideline is precisely that it IS evidence-based, and when people then come and argue, there's no point, I don't have any studies, although common sense says that it/they are aerosols, and if I sit with 30 children in too small a room without ventilation and masks, then they accumulate [..] the call for evidence is not always helpful, yes. Because that sometimes also blows wind into the sails of counter-arguments by simply claiming that there is no evidence” (B9, public health practitioner).

### Expertise and its role in the process

#### Understanding of expertise

The three notions of expertise present in the data with supporting quotes are displayed in Table 5, pointing to: scientific expertise grounded in scientific studies and disciplinary knowledge, practical expertise with implementing school and other measures and lived experience of being affected by school measures. It was also noted that the guideline secretariat had a decisive role in that they determined “who gets invited” (B8, scientist) and thus whose expertise was relevant for this guideline’s development. The convergence of these types of expertise as legitimate sources to inform recommendation development was described as rendering the composition of the guideline panel as appealing: “That was precisely what rendered the whole committee interesting, that everyone brought their professional and probably to a certain extent also their personal experience to the table” (B9, scientist).

**Table 5:** Sub-category “Characterisation and sources of expertise” containing the three notions of expertise present in the data with supporting quotes.

Type	Scientific expertise	Lived experience	Practical experience
Explanation	Expertise grounded in scientific studies and disciplinary knowledge	Expertise as lived experience of being affected by school measures	Expertise as practical experience with implementing school measures
Quotes	“So in principle, we tried to prove with this [long-standing professional experience and subject area knowledge] that we have expertise for this. Myself, I have been dealing with the topic of […] for decades […] and I would point to that to define what expertise for the topic means” (B6 scientist)“We are [not only] here because we are EbM [Evidence-based Medicine] specialists but because we adhere to a certain research tradition and we possess content-related expertise” (B5 scientist)	“I think, for me, expertise is when a person has gained an experience in a certain area […] and through that/ or has experienced it on their own body, […] That’s expertise for me” (B14, school family)“Surely personal experience, one’s own perspective [plays a role], clinicians assess things differently to scientists, affected individuals, parent representatives and student representatives […] have different experiences” (B9, scientist)	“I also try, when I notice something, to incorporate it. But actually, I’m more concerned with the daily problems. So the assessment. […] That’s not real/not evidence in the scientific sense. But definitely field reports” (B10, scientist practitioner)“… one’s own expertise, including one’s very subjective personal perspective which was formed during the first few months as I had very immediate and direct insights in certain areas of the pandemic process” (B4, scientist)

#### Role of expertise in the process

Expert opinion was considered crucial for the guideline development process, given the lack of evidence for many questions at hand, as well as to contextualise, interpret and, where appropriate, apply weightings to the available evidence. The perspectives of affected groups were also considered important, especially with regard to feasibility and practical implementation.

“I definitely need [..] the expertise of those who are somehow involved in the exact conceptualisation and implementation of measures. For example, the perspective from the school family: What can actually work? Or the perspective from the local health authority: How can I properly implement quarantine regulations in schools? In other words, scientific evidence must be coupled with anecdotal evidence or precisely with the expertise of the different affected groups” (B2, guideline secretariat).

The diversity of perspectives and opinions represented in the guideline panel was described as providing legitimacy for the guideline. However, one member of the school family pointed out that an even more diverse panel, e.g. with regard to age, would have reflected the diversity of the school family and school setting even better. An interesting reflection on the role of their own expertise was provided by this member of the school family: “I therefore knew what role we have, or what it meant that it is a medical guideline, where we as [professional title] can perform an advisory function. But also hold ourselves back concerning medical questions” (B13, school family).

#### Hierarchies in relation to expertise and degree of involvement

Participants pointed out that they perceived hierarchies based on seniority and academic credentials, with some scientists’ statements being more contested than others.

“I noticed that some of the statements made by [the person], although [the person] belongs to a scientific society that should actually have expertise in the field, were not so strongly accepted. On the contrary, they were often questioned, critically questioned. [..] in general, titles alone, no title, I perceived that to be making a difference in the process. [..] even if [..] a person is referred to as professor or doctor, I think that makes a difference in the discussion” (B8, scientist).

Whilst these differences in the recognition of expertise of members of different scientific societies were observed, even more striking perceived differences with regard to scientists versus members of the school family are reflected in the following statements.

“The parents [..] had to acquire such a comprehensive expertise [..] and yet we are not seen as experts because we might not belong to an association [..] are you an expert if you are a doctor or scientist and belong to an association [..] You are labelled, you are only such a small light and you do not belong to this big institution” (B11, school family).

“In a context with […] who all speak very scientifically, it sometimes is an effort to say something” (B14, school family).

Related to this, one participant argued that without proof of expertise in the form of relevant professional experience, a member of the school family should not be accepted as an expert: “[The person] also did not have anything in their CV based on which [the person] could have said I have expertise regarding this topic” (B6, scientist).

It was pointed out that a lack of experience with developing guidelines or similar products, with systematically appraising evidence as well as with using scientific language, not only, but specifically on the part of members of the school family, might have led to reduced possibilities for fully participating in the process.

“For me it was the first time that I took part in something like this and at the beginning it was perhaps also a little confusing and alienating [..] I mentioned at some point that many technical terms, abbreviations are very confusing for us because I then had the feeling that ‘Unfortunately I do not know what you are talking about’” (B11, school family).

Some also mentioned that they perceived the discourse to be strongly influenced by those members of the panel who were particularly “eloquent and in need of talking” (B10, public health practitioner), which left another participant rather “disillusioned [..] because I had the feeling that decisions were not primarily made on the basis of what was really known, but that in the discussion, for example, individuals who simply had a very high social standing due to their background or their position dominated very much [..], and that many people then simply followed this” (B8, scientist). Specifically, participants reflected on the role and influence of one institution during the process which was perceived as having a strong voice. Some participants even suggested that the guideline was therefore aligned with already existing recommendations:

“In various decisions, [the institution] has simply asserted itself. So many of the things that are currently in the guideline are actually points that [the institution] has in its school recommendations. And when the guideline wanted to deviate a bit from that, a veto was used until this compromise was reached again” (B7, guideline secretariat).

The weight of the students’ voices was described differently by different participants.

“It was not the case that one had the feeling that some scientists were dominating [..] the younger people from the school sector, for example. [..] there is a hierarchy gap; a chance, let's say, for this [the hierarchy gap] to have an effect. But in this process I had the feeling that it was actually going quite well, I have to say” (B5, scientist).

“The students who were very withdrawn. [..] I experienced them as very mute in the process [..] possibly because of these hierarchies that were not actively broken up” (B7, guideline secretariat).

A certain difference in self-perceived legitimacy amongst members of the school family versus those with a background in science could also be observed. This member of the school family was “very surprised when I was appointed. […] Because, as I said, S3 guideline did not mean anything to me. But I think it’s good that in this case, the three truly biggest players from the school sector were involved” (B12, school family). Conversely, this researcher explained that their organisation “was involved in this leading role from the very beginning. And we also discussed this internally in the board of directors that we [pseudonymised] also want to take on a leading role in the process” (B8, scientist). Contrastingly, some participants did not perceive any differences between themselves and others or within the panel at large and indeed emphasised that their specific expertise was sought and appreciated.

“In the beginning, what was new for me from the medical perspective was new for those from the practical school perspective. There was a process of convergence. But I did not feel that we were somehow second-class members of the expert council” (B12, school family).

“It is always mandated […] when it comes to medical guidelines, that there are also patients in it. But honestly, it is often a marginal contribution. And here it was not a marginal contribution. […] Everything that the academics contributed was countered a little bit by views from the world of the students, teachers, teachers' associations, parents” (B5, scientist).

### Consideration of societal implications and unintended consequences

#### Experience with applying the WHO-INTEGRATE framework

When participants were asked to recall how societal implications and unintended consequences of recommendations were considered, those who were part of the working groups or had a coordinating role elaborated on these in relation to the criteria laid out in the WHO-INTEGRATE framework. Participants noted, however, that unintended health consequences as well as social outcomes received a lot more attention relative to economic, ecological or legal aspects. It can also be assumed that the foci of consideration in the working groups varied, as pointed out by this participant:

“It happened in such a way that small groups suggested wordings, and these were then discussed. And that was very different. On the one hand, the level of wordings was different, the level of consideration of the different levels of the framework was very, very different” (B8, scientist).

Others, particularly members of the school family who were not part of the working groups, where the framework was explicitly applied, recalled that the weighing up of feasibility and further aspects happened in discussion during the full panel meetings (as opposed to a systematic examination of criteria). Crucially, it was remarked that “[the weighing up] was included through discussion. Although this is/ partly it is dependent on which persons/ who gets to talk” (B10, public health practitioner). From the perspective of members of the school family, the main tension to address when agreeing on recommendations was what was stipulated with regard to infectious disease control and what was desirable from an educational perspective (as opposed to a systematic, holistic consideration of various dimensions of unintended consequences). Participants noted that institutional interests likely also played a role in influencing participants’ lines of argumentation during these discussions.

#### Lack of evidence and expertise regarding unintended consequences and societal implications

It was noted by participants that both evidence and specific expertise for assessing societal implications and unintended consequences beyond direct health impacts was largely missing.

“As we didn't even have time to really discuss the advantages and disadvantages for health in such a way that I think everyone had their say as they should have done [..] the expertise was of course not in the group either. So [..] we didn't have anyone from that field. [..] should have been strengthened in any case by some expertise within the group” (B8, scientist).

Another participant noted that due to the lack of evidence, “good common sense” (B9, public health practitioner) played a big role in assessing any implications beyond health impacts as well as in agreeing on the strength of recommendations. Similarly, the lack of qualitative research regarding values or preferences of those affected by the recommendations was highlighted as a limitation.

#### Usefulness of applying an Evidence-to-Decision framework

Participants reflected on the relevance of EtD frameworks, especially in the context of developing guidelines during a public health emergency. In principle, and especially in the absence of conclusive evidence regarding the effectiveness of measures, they were perceived as being important to aid decision-making and to give a comprehensible structure to the process of arriving at recommendations, as it “helps us to create transparency […] in the justification of recommendations” (B3, guideline secretariat). Using an EtD framework was described to have helped the panel to ground their recommendations in reality and to explicitly consider any potential side effects of recommendations in a transparent manner according to this participant: “The appeal of the criteria [was] that we had a scheme that we worked with and that enabled us to make such considerations transparent, whereas in guidelines that do not use criteria, such things often feature in rather intuitively, implicitly, and are not made explicit” (B2, scientific secretariat).

Another participant, despite recognising the value of increased transparency, questioned the added value of using EtD frameworks in guideline development processes. This person questioned the usability for practitioners, as they had previously noticed “that they will not follow any more at some point. That they then say, this is actually too complex, too complicated for me” (B3, guideline secretariat). Based on this observation, they further questioned: “Are those really better guidelines, are they really better suited for practical considerations? Or are they better suited for the publication? […] I do not think we know that […] I am so far not aware that this results in the guidelines being better accepted and implemented” (B3, guideline secretariat). Another participant similarly questioned whether the working groups’ assessments of the WHO-INTEGRATE criteria were directly relevant for panel members’ voting behaviour or for the target group of the guideline.

## Discussion

The development of an S3-guideline to provide evidence- and consensus-based support for decision-makers regarding school measures during a public health emergency was unprecedented in Germany. The present study provides insights into the strengths and weaknesses of the guideline development process as perceived by the different groups involved.

### Key findings and implications

#### Role of the secretariat in developing this guideline

In addition to its overall coordinating role, the guideline secretariat systematically identified and appraised the evidence, provided methods support, notably by being involved in the application of the WHO-INTEGRATE framework in the working groups, and directly contributed to interpreting the evidence and drafting recommendations. It can be argued that this is justified by the considerable time pressure and the mixed levels of experience in guideline development and use of EtD frameworks, which warranted intensive support. As such, the secretariat’s work was appreciated by participants. Conversely, uniting too many tasks in one group can overburden this group and potentially lead to undue influence on the panel’s product. The tasks of interpreting the evidence and drafting recommendations are usually separate from the tasks of searching and synthesising evidence in well-established guideline development processes at the World Health Organization or the UK National Institute for Health and Care Excellence (15–17). Our findings emphasise the substantial temporal, human and financial resources as well as specific methods expertise required to develop evidence- and consensus-based public health guidelines. Adequate resource investment and specific expertise are likely needed regardless of whether public health guidelines are developed rapidly during a public health emergency or under normal circumstances.

#### Methods-related decisions in developing this guideline

The application of established AWMF procedures for clinical guidelines to the rapid development of a public health guideline happened during the ongoing process. Some methods-related decisions by the guideline secretariat in consultation with the scientific societies who formally registered the guideline were critically reflected upon by participants, suggesting that the pros and cons of these different decisions had not been made transparent. These included the selection of institutions invited to join the panel, the missing prioritisation of endpoints and the process of and lack of time allocated to the application of the WHO-INTEGRATE framework. As recommendations were developed in working groups composed only of scientists, the perspectives of those affected by or tasked with implementation of recommended measures were only included later in the process. Some participants perceived this separation of tasks as appropriate, whilst others felt that having all perspectives – representing different types of expertise as discussed below - included in the full process of recommendation development would add to their quality. Which of these approaches would be more time-efficient for a rapid guideline is unclear. This indicates that further methods-related reflection, nationally or internationally, and ideally agreement on which shortcuts are acceptable in guideline development during a public health emergency, is warranted with a particular focus on sources of bias (18).

#### Role and use of evidence

In line with AWMF procedures, the development of an S3-guideline required systematic searches for and quality assessment of scientific studies (7); the framing for what constituted reliable and valid scientific evidence was thus rooted in clinical medicine and public health. However, participants’ understanding of the concept and the characteristics of reliable and valid evidence varied, probably due to the different scholarly traditions of participants as well as insufficient time dedicated to building a common understanding at the beginning of the process. Furthermore, the body of evidence available to the panel was heterogeneous and characterised by various limitations (e.g. reliance on modelling studies, time lag between evidence production and use). Future (rapid) guideline development processes for public health questions would benefit from allocating more time to establishing a shared—and possibly more interdisciplinary—understanding of what constitutes valid and reliable evidence amongst panel members and those who support guideline development methodologically. This may help save time spent on resolving disagreements that stem from different epistemological standpoints.

#### Role and use of expertise

Panel members included those whose expertise should arguably be grounded in an understanding and critical assessment of scientific evidence, and those whose expertise was grounded in their lived experience. In the absence of a comprehensive and conclusive evidence base and under time pressure, those with a scientific background also had to draw from their own lived experiences during the pandemic. Nonetheless, whose expertise was accepted as such seems to have been contingent on proven professional experience, academic credentials as well as eloquence. To avoid undue influence of “convincing opinions” (19) especially in the absence of research evidence and to enable adequate consideration of all stakeholder’s perspectives, expert evidence—as opposed to expert opinion—should be used in a systematic and transparent way, including transparent procedures for collecting and appraising expert evidence to inform the process (19). Allocating sufficient time to establishing commonly agreed procedures for the consideration of different forms of expertise (from all parts of the spectrum described) as well as to training guideline-naïve panel members will be important for future public health guidelines, whether during emergencies or not. In light of the difficulties arising from the inclusion of different types of expertise, lack of experience with guideline development and a heterogeneous understanding of scientific evidence, methods-related reflections should also critically assess the benefit of including all affected stakeholders in rapid guideline development processes.

#### Use of the WHO-INTEGRATE framework

Health and societal implications of the recommendations were to be considered systematically by applying the WHO-INTEGRATE framework; they were further attended to by including representatives of those groups who would be concerned with or affected by the implementation of school measures in the panel. The expertise or “anecdotal evidence” of panel members regarding these aspects was the main source for these considerations given the lack of peer-reviewed studies as well as lack of professional expertise within the panel for some of the framework criteria which were also not formally prioritised and adapted to this guideline. However, the framework was mostly applied in the working groups of scientists with little time dedicated to the criteria in full group meetings that included practitioners and school family members; whilst health and educational consequences were discussed in these meetings, the framework’s criteria beyond these two areas were not usually systematically considered. The full potential of using this EtD framework might not have been harnessed given these limitations. It has been previously argued that EtD frameworks such as the GRADE EtD framework for health system and public health decisions can be readily used in rapid guidelines, too (20). Further applications of WHO-INTEGRATE in different types of public health guidelines and sharing lessons learnt could shed light on improved ways of using these framework’s procedures in general as well as for rapid guideline development processes.

### Strengths and limitations

#### Research framing and positionality

Making use of scientific evidence and following formal consensus processes amongst scientists and affected stakeholders has been established as the gold standard for producing guidelines by the World Health Organization as well as many national institutions across the world, including the AWMF in Germany (16, 21). This approach to developing recommendations forms part of the evidence-based medicine/evidence-based public health paradigm which the authors identify with. In the context of the present study, we did not question the evidence- and consensus-based approach to guideline development *per se* but examined the strengths and weaknesses of taking this approach to developing public health recommendations during a global health emergency.

#### Semi-external process evaluation

Three of the authors (EAR, LMP, BS) were members of the guideline secretariat and therefore potentially eligible to participate in this study; the two remaining authors (KW, MR) work in the same institution. The fact that this process evaluation was not conducted fully externally represents a main limitation of the study, as it may lead to social desirability bias in participants’ responses, or to selection bias in that participants holding strong positive or negative views regarding the guideline development process might be more likely to participate. Due to lack of dedicated funding, a truly independent evaluation of the guideline development process was not possible. We have been fully aware of this limitation from the start of the process and have sought to address any potential biases arising from it in the design and execution of the study. Importantly, recruitment, data collection and all but the very final steps of data analysis were solely carried out by KW and MR who also had exclusive access to the primary data.

#### Recruitment of participants

During recruitment we were actively seeking to reduce any selection bias that may arise from KW’s and MR’s potential knowledge of internal dynamics within the guideline development process; recruitment processes were exclusively performed by KW and MR. Instead of sampling participants purposively, all panel and secretariat members were approached and asked to express their interest in participating in the study. This process of self-selection might have led to the exclusion of potentially interesting perspectives of less extrovert or proactive individuals. Subsequently, random selection of participants amongst those who expressed their interest to participate was carried out. Representation of perspectives from all groups, including members of the school family, was achieved; however, the sample was not quantitatively balanced between the school family and those with a scientific background.

#### Data collection

During the interviews, acquiescence or desirability bias could have been present to not discredit the process or its output retrospectively. To counter this, we reiterated before the start of each interview that manuscripts would be fully anonymised, that potentially compromising statements would not be included in the analysis and that participants would be offered to review the transcript before analysis. Thus, we are relatively confident that participants did not refrain from disclosing their honest opinions and describing the process and interpersonal dynamics in rich enough detail to render this analysis meaningful. Recall bias might have been present as participants were asked to recount their experiences almost a year after the process had been initiated. Most of their answers referred to the first 3 months of developing recommendations for the short version of the guideline, although the interview questions did not stipulate this. No reflections on the difficulties and opportunities of developing “living guidelines” were elicited, however, we had also not included any probing questions about this in the interview guide.

#### Data analysis

Whilst the development of main categories was jointly undertaken by MR and KW, the development of sub-categories was carried out by one researcher (KW) only, therefore inter-coder reliability was not established for this step. However, KW established intra-coder reliability by reviewing and critically examining the sub-categories repeatedly over a period of 3 months. Data were rich and saturation in terms of included perspectives and the content of each category was likely achieved. Translating quotes from German into English, undertaken by KW and checked by EAR, was necessary to present our findings to an international audience, however this might have led to some loss or change of meaning. The researchers’ own background in public health and medical science might have influenced data analysis, particularly regarding the categories ‘Understanding of evidence’ and ‘Understanding of expertise’ as a certain shared understanding of these concepts is central to their epistemological standpoint and scientific socialisation. However, a process of reflecting about their own positionality accompanied the process of data collection and analysis and subjective interpretations were minimised by constantly scrutinising findings and their interpretation. Being close to the subject matter of qualitative research can also be seen as an advantage as it supports interpretation of the data in a way that is practically relevant. KW developed a first draft of the manuscript with a focus on choosing quotes that would not compromise confidentiality and yet provide a holistic account of the primary data. During review and subsequent refinement of the results, some aspects of the data presented were interpreted differently by the co-authors closely involved in the guideline process (EAR, BS and LMP). In these cases, KW presented further text passages and elaborated on their interpretation to ensure the analysis and conclusions were grounded in the data.

## Conclusion

The aim of producing an evidence- and consensus-based guideline on infection prevention and control in schools was to provide decision-makers and politicians with a salient basis for their decisions, rooted in scientific evidence and the expertise of a broad range of stakeholders, including scientists from across multiple disciplines, those tasked with the implementation of school measures and those affected by them. The rapid development of this public health guideline during a public health emergency was challenging, mostly due to enormous time pressure, with the first version of the guideline developed over the course of three  months only. More specific challenges were the limited evidence base, the lack of adequate financial and human resources as well as heterogeneity within the guideline panel and secretariat, notably regarding individuals’ understanding of reliable and valid evidence and experience with guideline development. The guideline development process was also characterised by some methods-related challenges, notably the use of a novel decision-making framework and the consultation of different types of expertise represented by a broad range of stakeholders beyond scientific societies. Learning from this process with a view to “institutionalising” the development of public health guidelines and refining public health-specific methodological approaches can contribute to more evidence-informed public health decision-making in Germany and beyond, in general and during a public health emergency.

## Data availability statement

The raw data supporting the conclusions of this article will be made available by the authors upon request, without undue reservation.

## Ethics statement

The studies involving human participants were reviewed and approved by Ethics Committee of the Medical Faculty, Ludwig-Maximilians-Universität München (No. 21-0944). Written informed consent to participate in this study was provided by the participants or their legal guardian/next of kin.

## Author contributions

EAR, BS and LMP conceived the overall research project. LMP, KW and MR jointly developed the protocol with substantial input from EAR and BS. Recruitment, data collection and all but the very final steps of data analysis were solely carried out by KW and MR who contributed equally to this research. All authors discussed the results and their implications. KW wrote the first draft of the manuscript. All authors critically reviewed the different versions of the manuscript, suggested revisions, and approved the version to be published.

## Funding

This research was undertaken without dedicated funding. Staff time was financially supported by the Chair of Public Health and Health Services Research at the LMU Munich.

## Conflict of interest

EAR, BS, and LMP are members of the guideline secretariat as well as initiators of this process evaluation, which represents a conflict of interest (see Methods and Discussion with regard to how this research sought to minimise any undue influence as a result of this conflict of interest).

The remaining authors declare that the research was conducted in the absence of any commercial or financial relationships that could be construed as a potential conflict of interest.

## Publisher’s note

All claims expressed in this article are solely those of the authors and do not necessarily represent those of their affiliated organizations, or those of the publisher, the editors and the reviewers. Any product that may be evaluated in this article, or claim that may be made by its manufacturer, is not guaranteed or endorsed by the publisher.
