# Supplementary material for: Rapid development of an evidence- and consensus-based guideline for controlling transmission of SARS-CoV-2 in schools during a public health emergency – A process evaluation
Source: Front Public Health. 2023 Mar 30;11:1075210. doi: 10.3389/fpubh.2023.1075210 (PMC10097910; doi:10.3389/fpubh.2023.1075210)
Supplement: Supplementary file 1 [file Table_1.docx]

# Annex 1: Interview guide (translated from German)

**Introduction**

Please briefly introduce yourself with your name and institution as well as your function in the guideline process.

Based on the introductory event, how did you understand your role within the guideline panel?

Were there any differences at different points during the guideline process?

**Process of deciding on recommendations**

How did you perceive the decision-making process regarding the recommendations within the guideline panel?

On what basis were the decisions made?

How did the decisions for the recommendations come about?

Were there any differences at different points during the guideline process?

**Understanding of evidence and expertise and their role in the process**

What role did evidence play in the guideline development process?

What do you understand by (scientific) evidence?

How is expertise to be understood in the context of producing a guideline?

To what extent do you feel that the recommendations are evidence-based?

Were there any differences at different points during the guideline process?

**Criteria for decision-making and their role in the process**

To what extent did other aspects or criteria besides evidence play a role?

How did you perceive the use of standardised criteria alongside evidence?

Which criteria played a particularly important role?

To what extent did these criteria influence the recommendations?

To what extent were positive and negative effects of the recommended measures considered?

Were there any differences at different points in the guideline process?

**Communication and consensus within the guideline panel**

How did you perceive communication within the guideline panel?

What is your view on the consensus-building procedures within the guideline panel?

To what extent did you feel that your opinion/expertise was considered?

Were there any differences at different points during the guideline process?

**Understanding of guidelines as an instrument of science-based policy advice**

In your view, how important was the guideline for political and practical decision-making during the pandemic?

How important was the guideline for politicians and other decision-makers from your point of view?

How important was the guideline for head teachers and the school family?

What can a guideline achieve during a crisis?

What can such a guideline not achieve?

**Lessons from the process and transferability**

What would you like to see in future guideline development processes?

What can be learned in principle from this guideline process for societal decisions?

What can be learned in principle from this guideline process for public health decisions?

**Closing**

To end, would you like to say anything else about the guideline?

Are there any aspects that have not been mentioned so far but which you think could play a role?

Do you think we forgot to ask anything that is important?
