# Supplementary material for: Rapid development of an evidence- and consensus-based guideline for controlling transmission of SARS-CoV-2 in schools during a public health emergency – A process evaluation
Source: Front Public Health. 2023 Mar 30;11:1075210. doi: 10.3389/fpubh.2023.1075210 (PMC10097910; doi:10.3389/fpubh.2023.1075210)
Supplement: Supplementary file 2 [file Table_2.docx]

# Annex 2: Deductively developed main categories with inductively developed sub-categories

| **Main categories** | **Sub-categories** | **Definition** | **Example quote** |
| --- | --- | --- | --- |
| **Perception of the guideline development process** |  | Includes the process of recommendation development within the guideline panel |  |
|  | Methods and implementation of the guideline development process | Statements about procedural and methods-related decisions to implement the guideline development process | „Whether I consider any evidence as meaningful or not is always a question of whether I assess the endpoint, how I assess it. We did not make a formal prioritisation of the endpoint. That was also a time problem. [...] So we presented and discussed them in the kick-off meeting so to say. But we did not formally prioritise them“ (B3, guideline secretariat) |
|  | Guideline development under time pressure | Statements regarding the difficulties that arose from time pressure | “that we were always sent the documents in a very short time, had to familiarise ourselves with them and then discuss them. In the discussion it became clear that not everyone had read everything. Which was hardly possible” (B8, scientist) |
| **Evidence and its role in the process** |  | Includes the interviewee's general understanding of evidence and the role of evidence in the process. |  |
|  | Understanding of evidence | Statements about the characteristics and sources of different types of evidence | “Scientific evidence for me is/ is based on a body of studies where the studies are planned and conducted in high quality, where the objectives are well formulated, where the methodology is correct. Yes, where one is guided by good scientific practice and independently and neutrally evaluates, interprets/analyses, interprets and correctly communicates results without being influenced by non-scientific aspects, or also not by political, I don't know, wishes, demands or whatever” B9, public health practitioner) |
|  | Role of evidence in the process | Statements about when and why different types of evidence played a role in the process | “That was really the starting point, I would say. Because the questions that needed to be answered, for those the available evidence was first of all substantially searched within the scientific literature, according to the rules of how this should happen.” (B5, scientist) |
| **Expertise and its role in the process** |  | Includes the interviewee's general understanding of expertise and the role of expertise in the process. |  |
| → | Understanding of expertise | Statements about the characteristics and sources of different types of expertise | “I think I have a broad understanding of expertise. That, for example, quite different groups of people can contribute expertise regarding the school context. Starting with the students, any caregivers, parents, educators, teachers, up to the (laughs) janitors and the authorities. And (...) at the same time, of course, they don't all have the same form of expertise, but perhaps constitute rather different facets that somehow come together to form a picture.” (B1, guideline secretariat) |
|  | Role of expertise in the process | Statements about when and why different types of expertise played a role in the process | “So in addition to the scientific evidence, the expert opinions have played a big role. This was mainly due to the fact that for many topics, we simply did not know the impacts.” (B8, scientist) |
| → | Hierarchies in relation to expertise and degree of involvement | Statements about the relational dynamics of individuals representing different types of expertise and their involvement in the process | “And the evidence based on the expertise of teachers and students and parents was not included in this process. They could just comment a bit afterwards. But I believe that they COULD have contributed aspects of feasibility or unintended consequences much better. But their expertise was not taken into account there, which could have potentially influenced the balance of the decisions” (B7, guideline secretariat) |
| **Consideration of societal implications and unintended consequences** |  | Includes the process of considering societal implications and unintended consequences in the process |  |
|  | Experience with applying the WHO-INTEGRATE framework | Statements regarding the consideration of societal implications and unintended consequences during the process | “Yes, there were always rounds of discussions about the extent to which a certain decision could have counterproductive effects” (B13, school family) |
|  | Lack of evidence and expertise regarding unintended consequences and societal implications | Statements regarding limitations of the process of considering societal implications and unintended consequences | “The expertise was of course not present in the group either. So, yes, no, we didn't have anyone from that area. And when they selected the groups, they made sure that we had people who were more concerned about the socially disadvantaged. Because that was very important to us. And I think we succeeded to some extent. But the other [areas] should have been strengthened by some kind of expertise within the group” (B8, scientist) |
|  | Usefulness of applying an Evidence-to-Decision framework | Statements regarding the value of using an Evidence-to-Decision framework | “And with the EtD framework, it's not clear to me yet whether that really leads to a/ except that it can be read more transparently and comprehensibly. Whether that really leads to an improvement” (B3, scientific secretariat) |
